# Synergistic Effect of Arbuscular Mycorrhizal Fungi and Germanium on the Growth, Nutritional Quality, and Health-Promoting Activities of *Spinacia oleracea* L.

**DOI:** 10.3390/plants13202869

**Published:** 2024-10-14

**Authors:** Basma Najar, Ahlem Zrig, Emad A. Alsherif, Samy Selim, Abeer S. Aloufi, Shereen Magdy Korany, Mousa Nhs, Mohammad Aldilam, Nahla Alsayd Bouqellah

**Affiliations:** 1 ULB—Faculty of PHARMACY, RD3—Pharmacognosy, Bioanalysis & Drug Discovery Unit & Analytical Platform of the Faculty of Pharmacy Bld Triomphe, Campus Plaine, CP 205/5, B-1050 Brussels, Belgium; 2 Dipartimento di Farmacia, Università di Pisa, 56126 Pisa, Italy; 3 Laboratory of Engineering Processes and Industrial Systems, Chemical Engineering Department, National School of Engineers of Gabes, University of Gabes, Gabes 6029, Tunisia; ahlem.zrig@fsg.u-gabes.tn; 4 Faculty of Sciences of Gabes, University of Gabes, Omar Ibn Khattab Street, Gabes 6029, Tunisia; 5 Botany and Microbiology Department, Faculty of Sciences, Beni-Suef University, Beni-Suef 62521, Egypt; emad_702001@yahoo.com; 6 Department of Clinical Laboratory Sciences, College of Applied Medical Sciences, Jouf University, Sakaka 72341, Saudi Arabia; sabdulsalam@ju.edu.sa; 7 Department of Biology, College of Science, Princess Nourah bint Abdulrahman University, P.O. Box 84428, Riyadh 11671, Saudi Arabia; asaloufi@pnu.edu.sa; 8 Botany and Microbiology Department, Faculty of Science, Helwan University, Cairo 11795, Egypt; shereen_abdelrehim@science.helwan.edu.eg (S.M.K.); mousanhs@aun.edu.eg (M.N.); 9 Biology Department, College of Science, King Abdulaziz University, Jeddah 21589, Saudi Arabia; maldilami@yahoo.com; 10 Biology Department, Science College, Taibah University, Almadina 42317, Saudi Arabia; nbouqellah@taibahu.edu.sa

**Keywords:** food quality, metabolite, antibacterial, antioxidant activity, germanium

## Abstract

Arbuscular mycorrhizal fungi (AMF) and the antioxidant germanium (Ge) are promising tools for boosting bioactive compound synthesis and producing healthier foods. However, their combined effect remains unexplored. This study demonstrates the synergistic impact of AMF and Ge on the growth, metabolite accumulation, biological activities, and nutritional qualities of *Spinacia oleracea* L. (spinach), a globally significant leafy vegetable. Individually, Ge and AMF increased biomass by 68.1% and 22.7%, respectively, while their combined effect led to an 86.3% increase. AMF and Ge also improved proximate composition, with AMF–Ge interaction enhancing crude fiber and mineral content (*p* < 0.05). Interestingly, AMF enhanced photosynthesis-related parameters (e.g., total chlorophyll) in Ge treated plants, which in turn increased carbohydrate accumulation. This accumulation could provide a route for the biosynthesis of amino acids, organic acids, and fatty acids, as evidenced by increased essential amino acid and organic acid levels. Consistently, the activity of key enzymes involved in amino acids biosynthesis (e.g., glutamine synthase (GS), methionine biosynthase (MS), lysine biosynthase (LS)) showed significant increments. Furthermore, AMF improved fatty acid levels, particularly unsaturated fatty acids in Ge-treated plants compared to the control. In addition, increased phenylalanine provided a precursor for the production of antioxidants (e.g., phenols and flavonoids), through the action of the enzyme phenylalanine ammonia-lyase (PAL), resulting in improved antioxidant activity gains as indicated by FRAP, ABTS, and DPPH assays. This study is the first to show that Ge enhances the beneficial effect of AMF on spinach, improving growth and nutritional quality, with promising implications for agricultural practices.

## 1. Introduction

Germanium (Ge), with a maximum concentration of only 7 mg/kg, is a common trace element in the Earth’s crust, mostly associated with minerals such as Zn, Pb, Cu, Sn, As, Fe, and Ag [1]. At a temperature greater than 250 °C, pure Ge slowly combines with air to generate germanium oxide. It dissolves readily in hot, strong acids but does not react with diluted acids. Ge tetrachloride and Ge tetraiodide are products of the reaction of germanium with halogens. Two oxides are produced when germanium and oxygen interact: germanium dioxide and monoxide. Additionally, it forms Ge telluride (GeTe), Ge diselenide (GeSe_2_), and sulfur disulfide (GeS_2_) [1]. Ge is safe when taken in food by amounts 0.4–3.4 mg, but organic forms of germanium may be UNSAFE when taken by mouth.

Studies have shown that germanium is present in almost all plants and animals [2]. Research by Lin Kuangfei et al. indicated that low quantities of germanium (2–8 mg·kg^−1^) increase soil urease activity, whereas higher concentrations decrease it [3]. Indoor and pot experiments revealed that Ge, at low concentration (10 mg·kg^−1^), enhanced soil catalase activity by 28.3% and 20.5%, respectively. Exposition to Ge also affects the content and composition of photosynthetic pigments in plants. Specifically, it impacts the photosynthetic pigments of algal species. For instance, treatment with 10 mg·L^−1^ germanium altered pigments in *Dicrateria zhanjiangensis*, *Dunaliella salina*, *Spirulina platensis*, and *Nannochloropsis* sp., with notable species-specific variations [4]. Carotenoids, the major pigments in algal cells responsible for light capture, can also be affected by germanium exposure [5,6]. Furthermore, research suggests that germanium may modify the pigments involved in algae photosynthetic processes by altering the Ge/Si ratio [7]. Plants containing Ge can enhance their antioxidant activity by forming complexes with antioxidant compounds. Xie et al. [8] synthesized quercetin–germanium (IV) complexes [(C_15_H_9_O_7_) Ge·2H_2_O] 2H_2_O, which demonstrated greater scavenging activity against hydroxyl radicals and superoxide anions than quercetin alone. Similarly, Bao et al. [9] found that rutin–Ge complexes were more effective than rutin alone at scavenging superoxide and DPPH radicals. Additionally, Provasoli [10] demonstrated that germanium forms stable chelates with oxalic acid, citric acid, and catechins, increasing antioxidant capacity and promoting plant health.

Arbuscular mycorrhizal fungi (AMF), belonging to the monophyletic *Glomeromycota* fungal lineage, establish intimate associations with most terrestrial plants worldwide. This symbiotic relationship involves the formation of extensive subterranean networks comprising hyphae and spores, which connect various plant species [11]. These associations play a crucial role in regulating terrestrial ecosystem functions and biodiversity by facilitating vital processes at the soil–plant interface, such as increased water and nutrient uptake, leading to improved plant nutrition [12]. Furthermore, AMF enhance stress tolerance, aiding in seedling establishment, growth, and survival [13], while also promoting plant succession by binding sand grains into larger aggregates, thereby improving soil structure [14]. Studies have shown that AMF assist host plants in thriving under low phosphorus (P) conditions and various stressors, including drought, salinity, herbivory, temperature fluctuations, heavy metal contamination, and diseases [15]. Overall, the symbiosis with AMF generally benefits plants by enhancing water absorption, productivity, and quality, thereby preserving soil fertility and stabilizing its structure [16]. These symbiotic interactions induce physiological, metabolic, and molecular changes in host plants, resulting in the accumulation of numerous secondary metabolites [17].

In the natural environment, Ge, a rare element in the Earth’s crust, typically coexists with either inorganic or organic substances. Natural soils contain inorganic Ge that can be absorbed and converted into organic Ge compounds by microbes, plants, and animals. Several natural organic Ge compounds can be found in medicinal plants such as ganoderma, ginseng, garlic, and *Angelica keiskei* Koidz [18]. Ge primarily influences the chlorophyll content in photosynthetic tissues and the enzyme activity in higher plants [19]. Additionally, germanium can preserve the integrity of the leaf membrane by preventing the production of harmful benzquinone and oxygen free radicals during the phenolic oxidation process in sunflower (*Helianthus annuus* L.) cells [20]. Pot experiments by Lin et al. [3] showed that low doses of Ge (<4 mg·kg^−1^) significantly enhanced chlorophyll, a content an in-rice leaves, while high concentrations (>15 mg·kg^−1^) dramatically reduced it. Similarly, Tang et al. [21], reported that GeO_2_ at a concentration of 5 mg·L^−1^ increased chlorophyll content in *Dendrobium officinale* Kimura & Migo pseudobulbs. Germanium also affects the nutritional content and chemical composition of plants. For instance, the addition of 400 mg·L^−1^ GeO_2_ to the medium increased the amino acid content of *Ganoderma lucidum* (Curtis) p. Karst mycelia’s rose by 54.34% [22]. Moreover, *Ganoderma applanatum* (Pers.) Pat. produced 36.62% more extracellular polysaccharides when exposed to 150 mg·L^−1^ of germanium compared to the control [22]. Wei Ming et al. found that the pseudobulb suspension culture of *Dendrobium huoshanense* Z. Z. Tang & S.J. Cheng had higher levels of reducing sugars and proteins when germanium (GeO_2_) was added to the culture media [23]. Tang et al. observed that GeO_2_ (5 mg·L^−1^) raised the soluble protein content in *Dendrobium officinale* pseudobulbs. [21].

Lian et al. demonstrated that soaking rice in GeO_2_ increased soluble sugar content and improved amylase activity, although extended soaking may be harmful to isoenzyme systems [24]. Ge can enter plant cells through active absorption and form Ge salts with organic carboxylic acids, thereby affecting certain cell enzymes [3]. Furthermore, Ge can increase the activity of both enzyme and non-enzyme antioxidants in plants. Wei et al. reported that adding 4.0 mg·L^−1^ GeO_2_ to *Dendrobium huoshanense* pseudobulb cells significantly decreased the glutathione (GSH)/oxidized glutathione (GSSG) ratios and GSH reductase (GSH) activity [23]. Ge was discovered to change peroxidase (POD) and catalase (CAT) activity in rice roots and leaves [25]. Low concentrations of Ge enhanced the activities of CAT, POD, superoxide dismutase (SOD), and glutathione peroxidase (GSH-Px) in rice [3]; conversely, high concentrations induced stress on the antioxidant enzyme system, leading to increased POD activity but decreased SOD and CAT activity. Lieu et al. [26] reported that Ge can promote plant growth and increase resistance to various stresses. Their review also highlighted that Ge application improved plant growth and stress tolerance in tomatoes and enhanced the yield and quality in crops such as wheat and rice. At low concentrations, Ge has been shown to enhance plants’ metabolism [27].

*Spinacia oleracea* L., commonly known as spinach, is one of the most significant and widely consumed leafy vegetables. It is not only inexpensive and widely available but also reputed for its medicinal properties and abundance of phenolic compounds and flavonoids [28,29]. Spinach’s low-calorie content, along with a rich supply of vitamins, micro- and macronutrients, and other phytochemicals, including phenols and fiber, contributes to its health-promoting qualities [30]. According to Nemadodzi et al. [31], *Spinacia oleracea* L. (spinach) is rich in nutrients beneficial to human health, including vitamins, minerals, and bioactive compounds. Its wealth of bioactive chemicals is associated with hypoglycemic, hypolipidemic, anticancer, and anti-obesity properties [32]. The combination application of AMF with other growth-promoting factors, such as biochar, has been shown to improve plant growth and metabolism [33]. Here, we thus hypothesize that AMF inoculation can enhance the nutritional value of spinach plants, particularly when combined with low concentrations of Ge. This combination was selected for their complementary benefits in improving plant growth and resilience. Ge enhances key metabolic processes, such as chlorophyll content, enzyme activity, and antioxidant defenses, while AMF boost nutrient uptake and stress tolerance through their symbiotic relationship with plant roots. Together, these treatments are expected to improve the growth, metabolism, and nutritional value of spinach plants, making them valuable for sustainable agricultural practices aimed at enhancing crop yield and quality.

## 2. Materials and Methods

### 2.1. Experiment Setup

At a water-holding capacity of 68%, sterilized soil and sterilized sand (Tref EGO substrates, Moerdijk, NL) were mixed at ratios from 70% to 30%. Two groups of soil were prepared: the first group received treatment with Rhizophagus irregularis, whereas the second group (control) received no treatment at all. Source: Agriculture Research Center, Giza, Egypt; source of spinach seeds. The seeds were immersed in 5% *v*/*v* sodium hypochlorite for 20 min to surface sterilize them. Initial tests on the growth response of spinach were conducted at different concentrations of Ge (GeO_2_) (0, 1, 5, 7.5, and 10 mg/L). Since 10 μg/mL has been shown to enhance plant growth by 35–40%, this concentration was selected for the primary experiment. The seeds were submerged in water or an aqueous GeO2 solution containing 5 mg/L for ten hours under the cover of darkness. Processed spinach seeds were grown in containers with soil from both groups—AMF-treated and untreated—that measured 10 cm in depth by 8 cm in diameter. The soil had a moisture content of 0.33 g water/g dry soil and the following nutritional status: carbon (11.7 mg), nitrate nitrogen (13.8 mg), ammonium-N (1.1 mg), and phosphorus (9.4 mg) per g of air-dried soil. Throughout the experiment, the soil water content (60%) was maintained, along with the EC of 3.4 dS/m, pH of 7.56, and K of 2.75 meq/L. Plants were grown in a climate-controlled laboratory with a temperature of 21/18 °C, 16/8 h of day and night, and a photoperiod of 150 μmol PAR m^−2^ s^−1^ at 60% humidity. The experimental design included a randomized full block design with five replicates for each treatment. Water from the tap has been used for routine irrigation. After 5 weeks, the fresh and dry biomass weights were measured, and plant samples were harvested, ground in liquid nitrogen, and stored at −80 °C for biochemical analysis. Soil samples were collected for use and stored at −20 °C for biochemical and physical analyses.

### 2.2. Photosynthesis-Related Parameters

The photosynthetic rate was calculated according to the method described by Lichtenthaler [34]. For pigment analysis, 200 mg of fresh sprouts was ground in liquid nitrogen and homogenized using a MagNALyser (Roche, Vilvoorde, Belgium) at 7000 rpm for 30 s in 5 mL of 95.5% acetone. After homogenization, the samples were centrifuged at 14,000× *g* for 20 min at 4 °C. The clear supernatant was filtered through an Acrodisc GHP filter with a pore size of 0.45 µm. Pigments such as lutein, carotene, and chlorophylls were evaluated using high-performance liquid chromatography (HPLC, Shimadzu SIL10-ADVP, Tokyo, Japan) with reversed-phase HPLC at 4 °C. The pigments were fractionated using a C18 silica column. The mobile phase consisted of acetonitrile, methanol, and water (81:9:10) as solvent A, and a mixture of methanol and ethyl acetate (68:32) as solvent B.

### 2.3. Determination of Ge Content

An amount of 0.5 g of the powdered material was transferred to a Falcon tube (25 mL) and digested with 10 mL of ultrapure-grade HNO₃ (Merck, Darmstadt, Germany) at 90 °C for 6 h. After digestion was complete, the residue was filtered through a Whatman No. 2 filter paper. The solutions were analyzed using ICP-MS or ICP-OES. Measured Ge content was assayed in triplicate, repeated three times, and expressed on a dry weight basis. Data quality was checked using Standard Reference Material 1574a (Peach Leaves) from NIST, USA.

### 2.4. Determination of the Nutritional Quality

Data on the proximate composition, mineral content, vitamins, phenolics, organic compounds, fatty acids, and amino acids were collected to assess the nutritional value of spinach plants.

#### 2.4.1. Analysis of Proximate Composition

The methodology outlined by Wong et al. [35] was utilized to determine the carbohydrate content of spinach plants, regardless of whether they had received AMF treatment or not. Additionally, the protein concentration (0.2 g FW) of every sample was ascertained by applying the Lowry et al. [36] technique. Following the instructions given by Shiva et al. [37], the samples were homogenized in a 2:1 (*v*/*v*) mixture of methanol and chloroform, and the total lipid content of the plants was assessed. Following a 15 min centrifugation at 3000× *g*, the plants were separated into pellets, which were dissolved in a 4:1 (*v*/*v*) ethanol and toluene mixture. The total lipid content was calculated by gravimetrically quantifying the recovered lipids and expressing the result as weight (mg) per fresh weight of sprout (g) after concentration. The procedure described by Lee et al. [38] was used to extract the crude fibers from the plants. The fibers were gelatinized for 25 min at pH 6 and 100 °C using heat-stable alpha-amylase. Then, using protease at pH 7.5 and 60 °C for 25 min and amyloglucosidase at pH 6 and 0 °C for 30 min, unwanted protein and starch were removed by enzymatic digestion. After being cleaned in ethanol, the fibers precipitated, and the leftover material was weighed.

#### 2.4.2. Elemental Analysis

Elemental measurement was applied using the procedure outlined by AbdElgawad et al. [39]. To determine the concentrations of potassium (K), phosphorus (P), calcium (Ca), magnesium (Mg), manganese (Mn), and zinc (Zn), 100 mg of dry powdered sprouts were subjected to a 25 min digestion process at 185 °C carried out in 13 M nitric acid. Inductively coupled plasma mass spectrometry (ICP-MS) was then used to perform the measurement, using a glass nebulizer (0.4 mL/min). The external standards were calibrated within the range of 1–600 μg/L using calibration curves, and 0.23 M nitric acid was used to prepare standard minerals.

#### 2.4.3. Levels of Amino Acids and Metabolism

The method described by Sinha et al. [40] was used to determine amino acid content. For quantification, 200 mg of sprout flour, made from pulverized sprouts, was homogenized in 80% ethanol and centrifuged at 22,000× *g* for 25 min. The supernatant was diluted and passed through a 0.2 μm-pore Millipore filter. The residue was subjected to a second round of extraction, centrifugation, and filtration. Amino acid levels in the filtrates were analyzed using the Waters Acquity UPLC-TQD system, with a low-pressure mobile phase of acetonitrile and water, and a detection at a wavelength of 250 nm. Results were expressed as milligrams of amino acids per gram of the sample’s dry weight. Glutamyl synthase (GS) activity was assessed using the method described by Almuhayawi et al. [41]. Dihydrodipicolinate Synthase (DHDPS) activity was quantified according to Kumpaisal et al. [42], while L-cystathionine formation was separated using the method described by Ravanel et al. [43].

#### 2.4.4. Organic Acid Analysis

Organic acids were determined by the method outlined by Hamad et al. [44]. High-performance liquid chromatography (HPLC) with a UV detection system set at 210 nm was used to identify and quantify organic acids. The quantification process involved comparing the obtained data with relevant standards. The detection system comprised an LED model detector (Ultimate 3000, Tokyo, Japan) and a liquid chromatographer (Dionex, Sunnyvale, CA, USA), equipped with an LPG-3400A pump, a TCC-3000SD column thermostat, and an EWPS-3000SI autosampler. The separation was conducted at 65 °C using an Aminex HPH-87 H (300 × 7.8 mm) column with an IG Cation H (30 × 4.6) precolumn from Bio-Rad company, (Hercules, CA, USA). Data analysis was performed using the Chromeleon v.6.8 computer program.

#### 2.4.5. Fatty Acid Analysis

For fatty acid identification via GC/MS, 200 mg of sprout samples were extracted; 250 mg of powdered sprouts was treated with 100% methanol at room temperature until the material lost color. Internal standards, such as nonadecanoic acids and codeine, were used throughout the extraction to ensure analytical accuracy. The GC/MS analysis was conducted using an Agilent Hewlett Packard (Hewlett Packard, Palo Alto, CA, USA) 6890 GC system with a 5975 Mass Selective Detector (MSD) and an HP-5ms column (30 m × 0.25 mm × 0.25 µm). Fatty acids were identified by comparing results with the Golm Metabolome Database (http://gmd.mpimp-golm.mpg.de) and the NIST 05 database. Quantification was achieved by comparing the peak area with the corresponding standard calibration curves.

#### 2.4.6. Determination of Vitamin Levels

Thiamine (vitamin B1, thiamine monohydrate, 420 nm), riboflavin (vitamin B2, 254 nm), and contents of plants were determined by HPLC and expressed as an mg/g FW method of Almuhayawi et al. [45]. Vitamin C (ascorbate) levels were determined according to the method by Farfan-Vignolo and Asard [46]. HPLC methods were used for quantifying vitamins. Fresh leaves were homogenized in 0.1 N HCl for the extraction of thiamine and riboflavin. Ascorbic acid (AsA) was extracted using meta-phosphoric acid and separated using a Polaris C18-A column (110 mm × 4.7 mm, 2.5 μm particle size) at 42 °C. Detection was performed using a diode array detector (DAD) (SPD-M10AVP, Shimadzu, Tokyo, Japan). Total AsA was determined after reduction with DTT (0.04 M). Tocopherols (Vitamin E) were extracted by homogenizing sprouts in hexane, separated using a Particil Pac 5 µm column, and measured using HPLC. An internal standard (5 ppm of 5,7-dimethyltocol) was used. Retinol (Vitamin A) was analyzed using reversed-phase HPLC on a silica-based C18 column, with a mobile phase of acetonitrile/methanol/water (81:9:10) and methanol/ethyl acetate (68:32). The solvent flow rate was 1.2 mL/ min, with detection by DAD at 420, 440, 462, and 660 nm. The concentration was determined using a calibration curve.

#### 2.4.7. Quantification of Phenolics and the Enzymes Involved in Their Biosynthesis

The antioxidants (phenolics and flavonoids) and antioxidant capacity (FRAP) were extracted in 80% ethanol. After centrifugation at 14,000× *g* for 25 min at 4 °C, the FRAP test [TPTZ (0.01 mM) in HCl (0.04 mM), acetate buffer (0.3 M, pH 3.6), and FeCl_3_·6H_2_O (0.02 M)] was conducted using Trolox (0–650 M) [45]. Briefly Phenylalanine ammonia-lyase (PAL) activity was determined using the method of Abd Elgawad et al. [39], in a reaction solution containing Tris-HCl (100 mM, pH 8.8) and L-phenylalanine (40 mM).

### 2.5. Biological Activities

#### 2.5.1. Antioxidant Activities

To determine the total antioxidant capacity, 100 mg of sprouts was shaken in 80% ethanol solution, followed by centrifugation at 14,000× *g* for 25 min at 4 °C. The FRAP (Ferric Reduction Antioxidant Power) assay was then performed, involving a reaction solution containing 0.3 M acetate buffer (pH 3.6), 0.01 mM TPTZ (2,4,6-tripyridyl-s-triazine) in 0.04 mM HCl, and 0.02 M FeCl_3_. The total antioxidant capacity was expressed as µmol Trolox equivalents per gram of plant extract using a standard curve. Additionally, the total antioxidant capacity was assessed spectrophotometrically by mixing extract supernatants with DPPH (2,2-diphenyl-1-picrylhydrazyl) reagent and measuring the absorbance at 517 nm.

#### 2.5.2. Antimicrobial Activity

The antimicrobial activity of the plant extracts was assessed using the conventional dilution method [41]. It was evaluated using the disc diffusion method, where the ethanol extracts of linseed sprouts were tested. A bacterial suspension (10^6^ CFU/mL) of the tested strain was spread over Muller–Hinton agar and extracted-loaded (10 g/disc) were placed on the agar plates. Ethanol-loaded discs served as negative controls while standard antibiotics were used as positive controls. After incubating the plates at 37 °C for 24 h, the inhibition zones were measured with Vernier calipers.

#### 2.5.3. Antidiabetic Activity

##### α-Amylase Inhibition Assay

To examine the α-amylase inhibitory effect, a modified method based on Dada et al. [47] was used. Sprout extract was mixed with a reaction solution containing starch (1 g/L) and phosphate buffer (pH 6.9). The reaction was initiated by adding 3 U/mL of amylase enzyme and stopped after 10 min of incubation by 0.5 mL of dinitro salicylic (DNS) reagent. The mixture was heated to 100 °C for 10 min, followed by the addition of 0.5 mL of a potassium sodium tartrate solution (40%). The absorbance was measured at 540 nm to determine the inhibitory activity.

##### α-Glucosidase Inhibition Assay

The α-glucosidase inhibition assay was performed by modifying the protocol described by Dada et al. [47]. The sprouts’ hydroethanolic extract was mixed with α-glucosidase (2 U/mL) and incubated at 37 °C for 5 min. After adding 1 mM para-nitrophenyl glucopyranoside dissolved in 50 mM phosphate buffer (pH 6.8), the reaction was incubated for 20 min at 37 °C. The reaction was halted by adding 1 M sodium carbonate solution. The generation of p-nitrophenyl glucopyranoside was measured via light absorption value at 405 nm, and the percentage inhibition of α-glucosidase activity was calculated using the following formula:

Percentage Inhibition = (Average A405 control − Average A405 extract)/(Average A405 control) × 100

##### Estimation of Glycemic Index

The glycemic index (GI) of the plant extract was estimated using in vitro starch hydrolysis as outlined by Brouns et al. [48]. Medicago sprouts were incubated for one hour at 40 °C in reaction solution containing HCl-KCl buffer (pH 1.5) and 100 mg/mL of pepsin. The mixture was then diluted in phosphate buffer (pH 6.9) and incubated with α-amylase at 37 °C. Aliquots (1 mL) were obtained every 30 min and boiled for 20 min to stop the enzyme activity. To convert the remaining starch to glucose, amyloglucosidase (60 µL) and 0.4 M sodium acetate buffer (pH 4.75) were added, and the mixture was incubated at 60 °C for 50 min. Aliquots (0.6 mL) were obtained and incubated for 37 °C, and the glucose content was measured.

### 2.6. Statistical Analyses

Statistical analyses were conducted using the SPSS program (SPSS Inc., version 22.0, Chicago, IL, USA). Data were subjected to one-way analysis of variance (ANOVA) to compare the effects of individual treatments (AMF or Ge) on the measured variables, allowing for the assessment of the variance between different treatment groups. Mean separations were determined using a post hoc Tukey’s multiple range test (*p* < 0.05). Cluster analysis based on Pearson’s distance metric was conducted on all parameters using the Mult Experiment Viewer (MeV 4.9.0) TM4 software package (Dana-Farber Cancer Institute, Boston, MA, USA).

## 3. Results

### 3.1. Impact of Various Treatments on Spinach Growth and Ge Accumulation

**Figure 1:**
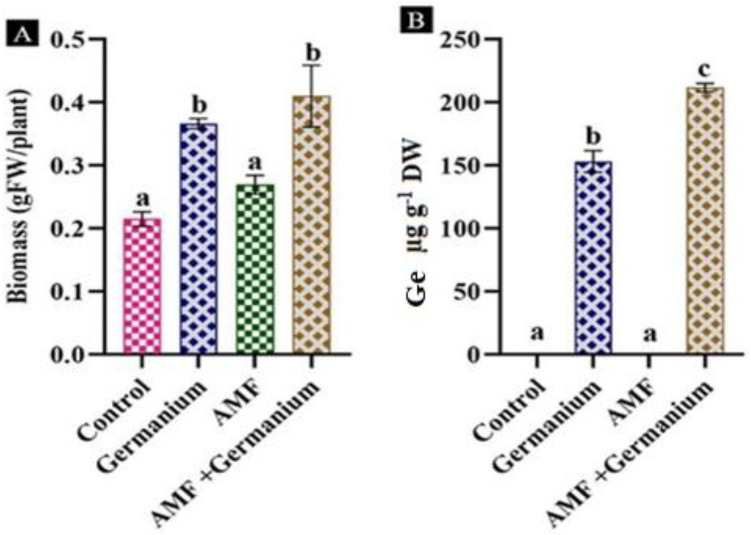
Effect of different treatments on biomass (**A**) and Ge level (**B**). Values expressed as means ± standard error of three independent replicates. Different letters above the bars, within the same organ, indicate significant differences between means at *p* = 0.05 using Tukey’s post hoc test.

In the present investigation, the application of Ge treatment induced an increase in fresh biomass accumulation of 68.1% in spinach (Figure 1A). AMF-inoculated plants also exhibited an increase in biomass by 22.7%. The combination of AMF and Ge led to the highest increase in biomass accumulation, with an 86.3% increase compared to the control plants. The application of Ge treatment induced an increase in Ge concentration in spinach plant (150 µg g^−1^), while the combination of AMF and Ge led to an increase in Ge more that the treatment by Ge alone by 40% (Figure 1B).

### 3.2. Photosynthetic Pigment

**Figure 2:**
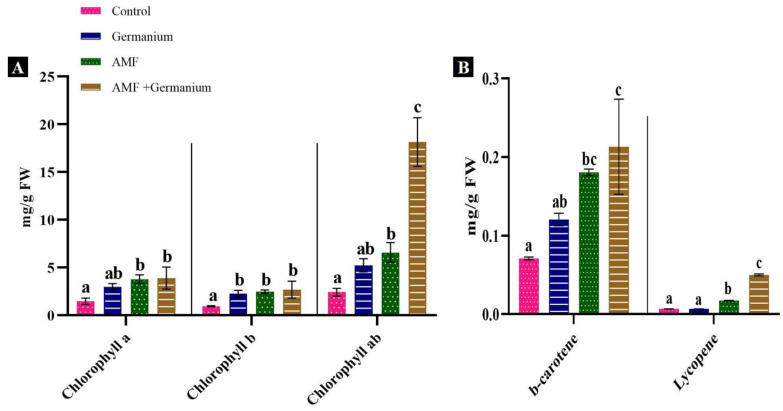
Effect of different treatments on photosynthetic pigments, (**A**): chlorophyll a, chlorophyll b and chlorophyll ab; (**B**) b-carotene, lycopene. Values expressed as means ± standard error of three independent replicates. Different letters above the bars, within the same organ, indicate significant differences between means at *p* = 0.05 using Tukey’s post hoc test.

The effect of combined AMF and Ge priming on the photosynthetic pigment of spinach plants was investigated. The results showed that the treatments significantly enhanced chlorophyll a, chlorophyll b, total chlorophyll a + b, beta-carotene, and lycopene levels compared to the control (Figure 2). The Ge treatment alone increased these photosynthetic parameters to levels lower than those observed with AMF alone. AMF treatment alone increased chlorophyll a by 156.4%, chlorophyll b 160%, total chlorophyll by 171.9%, beta-carotene by 157.1%, and lycopene by 200% (*p* < 0.05). The combined AMF and Ge treatment caused the highest increases in chlorophyll a (165.3%), chlorophyll b (184.2%), and total chlorophyll (649.5%) (Figure 2A). In addition, beta-carotene and lycopene increased by 200% and 400%, respectively (Figure 2B).

### 3.3. Effect of Ge and AMF on the Proximate Composition, Minerals and Vitamins of Spinach Plant

**Figure 3:**
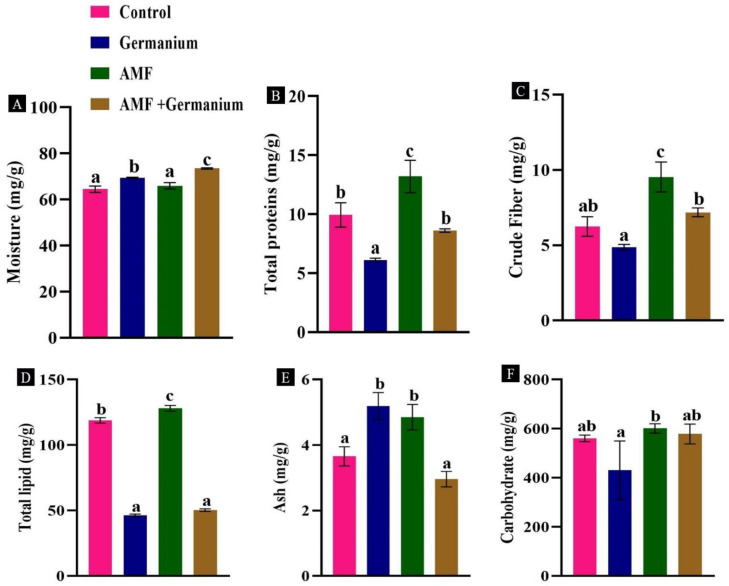
Effect of different treatments on proximate composition, (**A**): moisture, (**B**): total protein, (**C**) crude fiber, (**D**): total lipd, (**E**): ash content, (**F**): carbohydrates. Values expressed as means ± standard error of three independent replicates. Different letters above the bars, within the same organ, indicate significant differences between means at *p* = 0.05 using Tukey’s post hoc test.

The effect of Ge and AMF colonization by mycorrhizae on the proximate composition of spinach plant was assessed, including total protein, fat, ash, crude fiber, and carbohydrate contents (Figure 3). AMF-treated plants showed significantly higher contents of total protein (32.7%), fat (7.7%), crude fiber (52.8%), ash (32.7%), and carbohydrate (7.1%) compared to the control samples (*p* < 0.05). Under Ge and the combination of Ge and AMF treatments, moisture content also slightly increased by about 2.2% compared to the untreated plants (Figure 3A). The combination Ge and AMF treatment decreased protein by 13.3% (Figure 3B), increased crude fiber by 15% (Figure 3C), and decreased fat and ash contents by 56.5% and 19.1%, respectively (Figure 3D,E). Ge treatment alone increased all these parameters except carbohydrates, which declined by 23.2% (Figure 3F) compared to the untreated plants.

**Figure 4:**
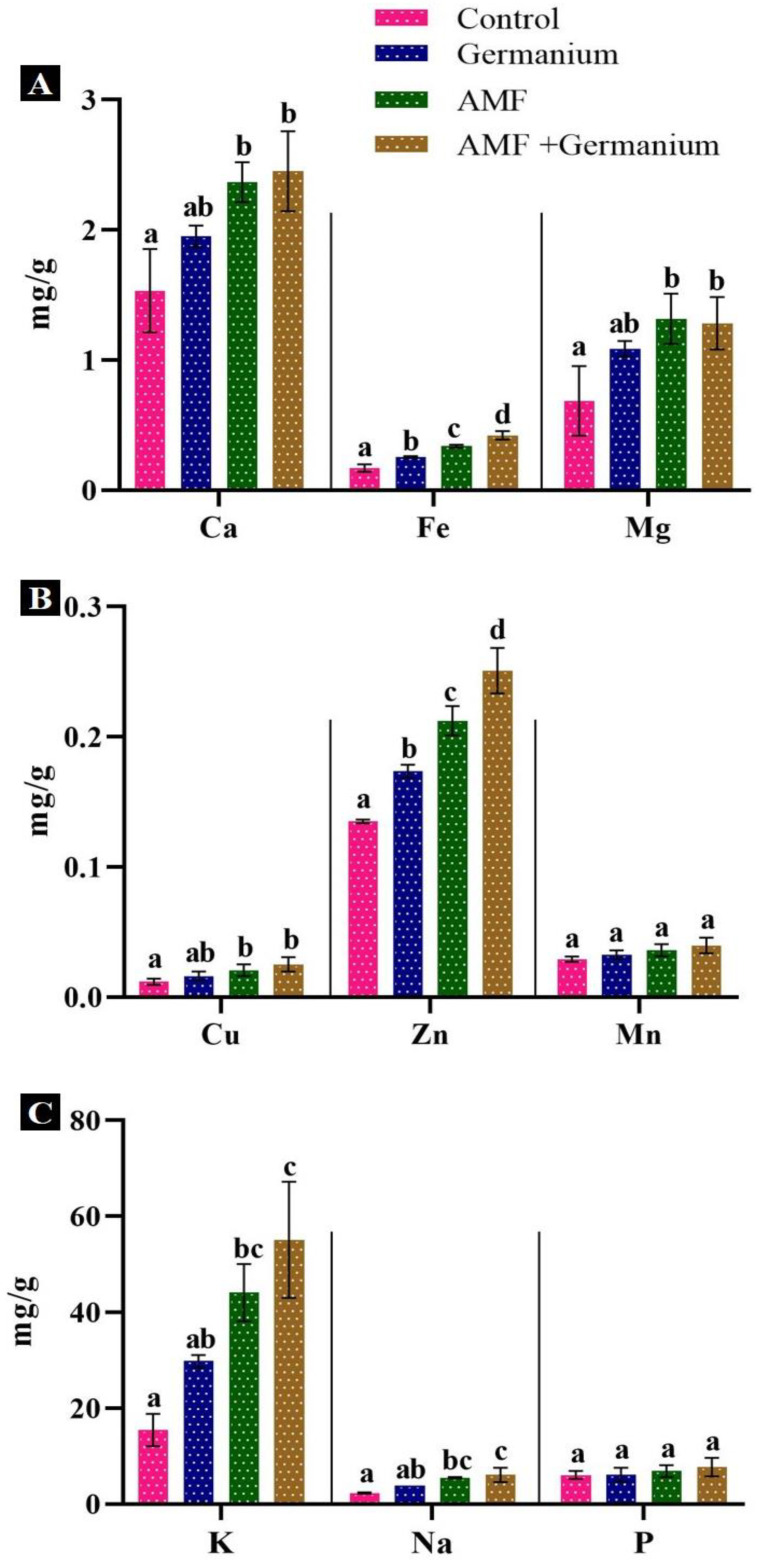
Effect of different treatments on mineral contents, (**A**): Ca, FE and Mg; (**B**): Cu, Zn and Mn (**C**): K, Na and P. Values expressed as means ± standard error of three independent replicates. Different letters above the bars, within the same organ, indicate significant differences between means at *p* = 0.05 using Tukey’s post hoc test.

Concerning mineral contents, AMF-treated plants, Ge-treated plants, and their combined-treated plants displayed significantly higher contents (*p* < 0.05) of all measured minerals (Figure 4). Magnesium (Mg) and phosphorus levels increased by 91.3% and 30.8%, respectively, and iron (Fe) content increased by 100% (Figure 4A). Zinc (Zn) and manganese (Mn) levels increased by 61.5% and 33.3%, respectively, in AMF-treated plants in comparison with the untreated plants (Figure 4B). AMF treatment alone resulted in the highest increases in potassium (K), sodium (Na), and phosphorus (P) by 159.4%, 139%, and 30.8%, respectively (Figure 4C). Ge treatment led to increased content of all measured elements, with the highest increases in K and copper (Cu) by 92.4% and 200%, respectively. The combined Ge and AMF treatment resulted in the highest increases in all studied minerals.

**Figure 5:**
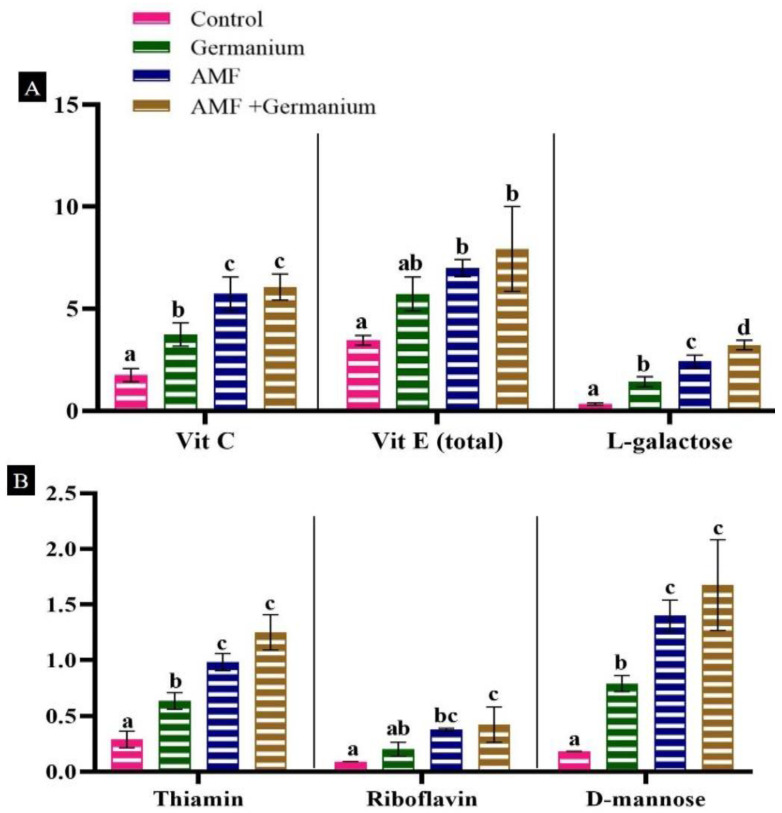
Effect of different treatments on vitamin and sugar contents, (**A**): vitamin C, vitamin and L-galactose; (**B**): vitamin B1, vitamin B2 and D-mannose. Values expressed as means ± standard error of three independent replicates. Different letters above the bars, within the same organ, indicate significant differences between means at *p* = 0.05 using Tukey’s post hoc test.

When examining vitamin content, Ge treatment alone, AMF treatment alone, and the combination of both treatments significantly increased the levels of all vitamins (Figure 5). AMF-treated plants showed the highest increases in vitamin C, vitamin E, thiamine, riboflavin, D-mannose, and L-galactose (*p* < 0.05) by about 227%, 102.8%, 237.9%, 322.2%, 677.7%, and 617.6%, respectively compared to the control (Figure 5A,B). While Ge treatment increased in the examined vitamins, it generally showed lower increases than those observed with AMF, except for vitamin E. The combined treatments resulted in the highest increases in all the studied vitamins.

### 3.4. Impact of Ge and AMF Treatments on the Functional Food Potentiality of Spinach Plants

To investigate the impact of Ge and AMF treatments on the functional food potential, nutritional quality and bioactive metabolites of spinach plants, the levels of amino acids (Table 1), fatty acids (Table 2), and organic acids (Table 3) in both the treated and untreated plants were assessed. Table 1 showed that eighteen out of twenty-two examined amino acids significantly increased in the treated plants when exposed to Ge, AMF, or their combination compared to the control plants (*p* < 0.05). Isoleucine, tryptophane, and arginine showed the highest increments under Ge by 158.9%, 102.4%, and 122.8%, respectively. Under the combination of AMF + Ge treatment, these amino acids increased further by 245.3%, 134.3%, and 213.5%, respectively (*p* < 0.05). Consistently, the activity of glutamine synthase (GS), methionine biosynthase (MS), and lysine biosynthase (LS) enzymes involved in amino acids biosynthesis showed significant increments (*p* < 0.05). However, some amino acids, such as histidine, alanine, valine, and leucine, showed increases, while glycine, methionine, and phenylalanine showed reductions under AMF treatment (Table 1).

Ge treatment also led to an increase in malic, succinic, citric, and lactic acids, with malic acid showing the highest increase at 287.2% and citric acid at 15.8% (Table 2). In contrast, oxalic acid decreased by 21.37%. The five estimated organic acids recorded increases in AMF-treated plants, with citric acid exhibiting a non-significant increase at 13.6% and succinic acid showing an increase by 5.5% (Table 2). The combined Ge+ AMF treatment increased oxalic, malic, and citric acids, with malic acid showing the highest increase. In contrast, the combined treatment reduced succinic and lactic acids by 2.1% and 14.3%, respectively.

Similarly, all detected fatty acids in the target plant were promoted when inoculated with AMF compared with the control plants (Table 3). One fatty acid, pentacosanoic (C24:0), decreased by 13.9% under Ge treatment, while the other fatty acids showed increases ranging from 12.6% and 276.4%. The combined treatment caused an increase in eleven fatty acids out of the fifteen (ranged between 2.7% and 271%), while the other four fatty acids exhibited decreases ranging from 3.2% (pentadecanoic (C16:0)) to 17.8% (octadecanoic (C18:2)). Octadecanoic (C18:1) showed the highest increases under Ge treatment and the combined Ge + AMF treatment, by 276.4% and 242.9%, respectively.

### 3.5. AMF and Ge Enhanced the Biological Activities of Spinach Plants

#### 3.5.1. Antioxidant Activity

To evaluate the impact of AMF and Ge treatment on the overall antioxidant activity of spinach plants, total phenolics, total flavonoids, FRAP, ABTS, and DPPH were determined in the treated and untreated plants (Table 4).

The increases in phenolic contents in response to Ge and AMF were 147.7% and 4.4%, respectively. Ge treatment alone caused increases in total antioxidant activity, as represented by total phenolics, total flavonoids, FRAP, ABTS, and DPPH by approximately 147.7%, 50.6%, 92%, 145.7%, and 27.3%, respectively, compared to the control plants (*p* < 0.05). In contrast, AMF treatment caused a slight increase in total phenolic compounds and a substantial increase in DPPH by 470.9% but decreased both flavonoids and FRAP by 13.6% and 56.4%, respectively. The combined effect of Ge + AMF increased total phenol, flavonoid, ABTS, and DPPH, while it caused a decrease in FRAP contents by 6.8%.

#### 3.5.2. Antimicrobial Activity

The results clearly demonstrate that Ge treatment significantly improved the antimicrobial activity of spinach plant against *Aspergillus flavus*, *Streptococcus salivarius*, *Salmonella typhimurium*, and *Candida albicans* by 35.3%, 26.6%, 24.8%, and 14.3%, respectively (*p* < 0.05) (Table 5). AMF treatment also induced antibacterial properties against a range of fungi and bacteria, with the most potent effect observed against *Aspergillus flavus*, showing an increase of 38.5%. The combined AMF + Ge treatment led to a significant increase in the antimicrobial activity against *Aspergillus flavus* and *Salmonella typhimurium* by 46.9% and 31.3% respectively.

#### 3.5.3. Antidiabetic Activity

**Figure 6:**
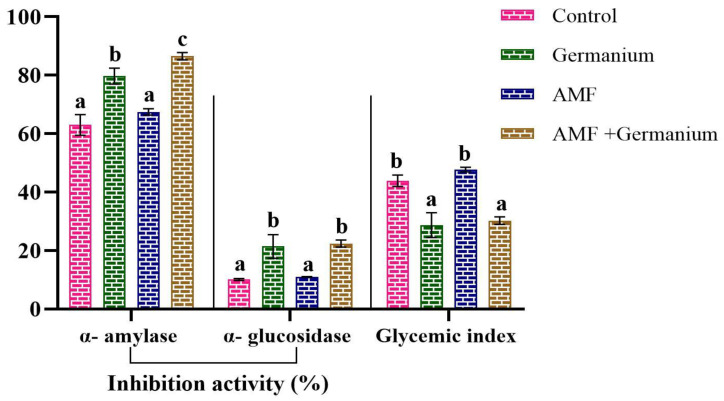
Effect of different treatments on antidiabetic activity. Values expressed as means ± standard error of three independent replicates. Different letters above the bars, within the same organ, indicate significant differences between means at *p* = 0.05 using Tukey’s post hoc test.

Notable increases in antidiabetic activities were observed in AMF-treated plants, as indicated by the increases in α-amylase inhibition activity (7.05%), α-glucosidase inhibition activity (8.6%), and glycemic index (8.7%) (*p* < 0.05). Ge treatment resulted in a substantial rise in α-amylase inhibition activity (26.4%) and α-glucosidase inhibition activity (111.3%), while the glycemic index decreased by around 34.5% (Figure 6).

## 4. Discussion

### 4.1. How AMF Improved Ge Effect on Spinach Plant Growth?

The current finding proved that AMF further improved Ge-induced spinach growth.

Puerner et al. [49] found that Ge increases the biomass and root length of treated plants. [49]. In their review, Liu et al. [26] stated that GeO_2_ treatment increases root activity and promotes the root growth and development of maize (*Zea mays* L.). Additionally, prior research has shown that Ge can promote seedling growth in vegetables, grains, and medicinal plants [50]. The synergetic effect of AMF and Ge can be explained by the enhanced ability of AMF to absorb vital nutrients, including P and Ge, which in turn stimulates plant growth and biomass production. Previous studies have indicated that AMF can access even the smallest soil pores, thereby promoting plant growth and enhancing nutrient and water absorption, ultimately leading to increased crop yields [51]. For instance, according to Zhang et al. [52], AMF facilitate the uptake of minerals (P, K, Fe, Zn, and Mn) by plant root. In this context, treating AMF enhanced nutrient uptake from organic fertilizers by stimulating acid and alkaline phosphatase activity [53]. This, in turn, facilitated phosphorus acquisition, boosted photosynthesis, and promoted overall plant growth. Similar to essential elements, Ge at low concentrations can also play a role in promoting plant growth through improving crop quality by boosting nutritional content, thereby improving the quality of fruits and vegetables [27].

The ability of AMF to increase photosynthesis could also explain the improved growth and nutrition value [53]. AMF allows the plant to access water more efficiently, to promote higher chlorophyll content, improving light capture and energy conversion which supports photosynthetic function [54]. Exposure to Ge also improved the content and composition of photosynthetic pigments in plants [4]. Photosynthesis is the main driver of biomass accumulation and provides the carbon skeleton for the biosynthesis of all metabolites [55]. Thus, the observed improvements in biomass accumulation could be attributed to the higher C assimilation under both AMF and Ge treatments. Supportively, we show that AMF and Ge have a synergistic effect on photosynthesis-related parameters, boosting more biomass accumulation.

### 4.2. Mycorrhizal Colonization Improved Tissue Chemical Composition of Ge-Primed Spinach

Improved photosynthesis increased sugar accumulation, directing more sugars to the roots and boosting sink strength [56]. AMF colonization also enhanced source-to-sink gene expression and sugar transport [57], correlating higher sugar levels with increased metabolic activity in treated plants. In *Dendrobium huoshanense*, an optimal concentration of Ge was found to increase intracellular soluble sugar levels and improved amylase activity [24]. Moreover, it has been documented that Ge can form complexes with natural sugars, including sucrose and mannitol, as well as with natural acids [58]. This suggested that Ge can improve plant cell functions, enhance carbon and nitrogen uptake, and accelerate protein and carbohydrate synthesis [23]. Sugars like glucose and sucrose are vital structural and energy compounds in higher plants [59]. As the main molecules transported from photosynthetic tissues to sinks, soluble sugars provide energy and essential building blocks for growth and function [60]. A significant portion of sugars is used for the cell wall biosynthesis in developing tissues [61].

High sugar accumulation under AMF and Ge growth conditions may supply carbon skeletons for other primary metabolites such as amino acids, fatty acids, and organic acids. In agreement, at low concentrations, Ge has been shown to enhance plant metabolism [27]. In this regard, Pepe et al. [62] found a strong correlation between the enhanced mineral uptake of mycorrhizal plants and their elevated levels of bioactive compounds. This improvement also enhances photosynthesis, leading to increased carbohydrate accumulation during nighttime respiration. Both Ge and AMF showed an ability to increase the number of polysaccharides [22], amino acids [63], respiration [64], oxidase activity [65], and its ability to promote plant cell growth [23]. Previously, it was reported that AMF, through their symbiotic connections with plant roots, positively impacted numerous aspects of plant chemistry, including fatty acid composition, organic acid content, and amino acid composition [66]. At metabolic levels, we observed that increases in the activity of lysine biosynthase (LS) and methionine biosynthase (MS) play critical roles in the synthesis of lysine and methionine. The accumulated amino acids play a pleiotropic role in plant physiology and metabolism, where they reserve as an energy storage as well as are involved in maintaining the osmotic potential of the plant cell, protecting membranes and stabilizing the photosystem I [67].

The accumulation of amino acids, such as phenylalanine, plays a significant role in antioxidant production [67]. PAL is a crucial enzyme in plant metabolism, responsible for catalyzing the conversion of phenylalanine into trans-cinnamic acid, the first step in the phenylpropanoid pathway. This pathway leads to the production of a variety of important secondary metabolites, including phenolics [67], which have antioxidant properties [68]. Here we found that this enzyme activity was increased by AMF and Ge. Similarly, it was previously found that AMF increased PAL activity [67]. Ge also not only neutralizes free radicals to protect healthy physiological metabolism, but it also modifies the function of antioxidant systems in both fungi and plants, which could be attributed to the improvement of AMF activities. Dang et al. [63] discovered that adding 400 mg·L^−1^ GeO_2_ to the medium increased SOD activity in *Ganoderma lucidum* mycelia by 16.73%. Ge could increase the activity of both enzyme and non-enzyme antioxidants in plants including glutathione and its related antioxidant enzymes [23]. Ge also enhanced POD and CAT activity [3,25]; conversely, high concentrations induced stress on the antioxidant enzyme system, leading to increased POD activity but decreased SOD and CAT activity. Their review also highlighted that germanium application improved plant growth and stress tolerance in tomatoes and enhanced yield and quality in crops such as wheat and rice.

### 4.3. Spinach Exhibited Enhanced Biological Activity Following AMF Colonization and Ge Priming

AMF has been demonstrated to significantly boost a plant’s biological activity, facilitating the nutrient exchange between the two partners, which offers several benefits, including the accumulation of bioactive metabolites. Similarly, at low concentrations, Ge has been shown to enhance plant secondary metabolism, promoting the production of valuable compounds such as phenolics and flavonoids, which are vital for improved plant biological value [27]. As a result, spinach’s antioxidant activity rose following AMF inoculation and Ge [60]. Similarly, Juge et al. [68] found that fungal symbiosis increased soybean antioxidant activity. Including Ge and AMF-treated spinach plants in the diet could be valuable for individuals looking to manage blood sugar levels. Overall, the antidiabetic, antibacterial, and antioxidant properties of spinach, coupled with its nutritional value, make it a beneficial component of a balanced diet aimed at promoting wellness and preventing chronic diseases.

At the antimicrobial activity level, recently, Huda-Faujan et al. [69] reported that antioxidants from spinach extract have potential applications in food preservation, as they can prevent cellular oxidative damage, improve storage stability, and limit the growth of various pathogenic bacteria. The focus of this work was on the potent antibacterial and antioxidant qualities of the phytochemicals found in spinach extract. In addition, Kadam et al. [70] found that mouthwash containing *Spinacia oleracea* extract could be used as a herbal alternative to conventional mouthwash, as it is equally effective against bacteria such as *Lactobacillus acidophilus*, *Porphyromonas gingivalis*, and *Streptococcus mutans* when compared to mouthwash containing 0.2% chlorhexidine. This synergistic effect not only enhances nutritional value but also adds therapeutic value. The antioxidant, antidiabetic, and antibacterial properties of AMF- and Ge-treated spinach present promising applications in both health and food preservation, reinforcing its role in promoting wellness and preventing chronic diseases.

## 5. Conclusions

The synergistic effects of Ge and AMF did not only improve biomass accumulation, but also primary (sugars, essential amino acids, fatty acids, and organic acids) and secondary metabolites. Furthermore, the antimicrobial and antidiabetic activities of Ge- and AMF-treated spinach plant were induced, making them a potent functional food. Future studies should explore the specific biochemical molecular mechanisms through which AMF and Ge interact. Moreover, Ge and AMF effects across different plant species and growing conditions should be investigated to fully harness their agricultural benefits.

## Figures and Tables

**Table 1:** Effect of different treatments on amino acid concentrations.

Amino Acids (mg/gFW)	Control	Germanium	AMF	AMF + Germanium
Aspartic (Asp)	11.39 ± 0.74 a	13.21 ± 0.13 b	12.72 ± 0.04 ab	14.19 ± 0.15 b
Glutamic (Glu)	17.21 ± 0.12 a	27.71 ± 1.39 b	18.79 ± 0.13 a	31.35 ± 1.51 b
Glutamine (Gln)	14.52 ± 0.38 a	19.14 ± 0.64 b	15.63 ± 0.09 a	21.35 ± 0.69 b
Serine (Se)	8.00 ± 1.95 a	10.46 ± 1.92 a	9.22 ± 1.48 a	13.15 ± 2.09 a
Glycine (Gly)	7.28 ± 0.73 a	10.09 ± 0.20 b	7.12 ± 0.75 a	11.13 ± 0.22 b
Arginine (Arg)	14.02 ± 2.57 a	37.26 ± 10.74 a	15.14 ± 1.34 a	43.97 ± 11.64 a
Alanine (Ala)	3.33 ± 0.21 a	2.67 ± 0.38 a	3.83 ± 0.14 a	3.26 ± 0.42 a
Histidine (His)	5.30 ± 0.42 a	4.21 ± 0.53 a	6.00 ± 0.18 a	4.07 ± 0.57 a
Valine (Val)	7.63 ± 0.83 a	4.67 ± 1.95 a	8.98 ± 0.32 a	3.23 ± 2.11 a
Methionine (Met)	2.02 ± 0.25 a	3.63 ± 0.19 b	1.93 ± 0.20 a	3.75 ± 0.20 b
Cystine (Cys)	1.51 ± 0.05 a	2.97 ± 0.34 b	1.69 ± 0.05 a	3.54 ± 0.36 b
Isoleucine (Ile)	4.44 ± 0.62 a	11.50 ± 3.05 ab	4.62 ± 0.16 a	15.33 ± 3.31 b
Leucine (Le)	13.74 ± 0.77 a	12.98 ± 1.01 a	15.04 ± 0.29 a	13.12 ± 1.09 a
Tyrosine (Tyr)	6.20 ± 0.27 a	7.99 ± 0.072 b	6.63 ± 0.22 a	8.73 ± 0.07 b
Lysine (Lys)	14.10 ± 0.44 a	25.22 ± 4.60 ab	15.45 ± 0.07 a	31.66 ± 4.99 b
Threonine (Thr)	4.55 ± 0.23 a	5.49 ± 0.68 a	5.19 ± 0.20 a	6.60 ± 0.74 a
Tryptophane (Trp)	0.36 ± 0.01 a	0.74 ± 0.05 b	0.39 ± 0.003 a	0.85 ± 0.06 b
Glutamine synthase (GS)	6.34 ± 0.08 a	8.37 ± 0.67 ab	6.55 ± 0.33 a	9.54 ± 0.65 b
Lysine biosynthase (LS)	2.36 ± 0.38 a	2.97 ± 0.54 a	2.54 ± 0.19 a	3.73 ± 0.59 a
Methionine biosynthase (MS)	0.02 ± 0.00 a	0.03 ± 0.00 b	0.02 ± 0.00 a	0.04 ± 0.00 b
Phenylalanine (Phe)	11.73 ± 2.01 a	21.73 ± 2.01 b	10.56 ± 1.72 a	21.40 ± 1.72 b
Phenylalanine aminolyase (PAL)	3.35 ± 0.28 a	5.54 ± 0.53 b	3.51 ± 0.04 a	6.28 ± 0.33 b

The lowercase letters (a and b) indicate statistically significant differences between the samples. The statistical significance of the relative abundances was determined by Tukey’s post hoc test with *p* < 0.05.

**Table 2:** Effect of different treatments on organic acids.

Organic Acid (mg/gFW)	Control	Germanium	AMF	AMF + Germanium
Oxalic	2.05 ± 0.11 a	1.75 ± 0.25 a	2.18 ± 0.05 a	2.37 ± 0.30 a
Succinic	7.93 ± 0.55 ab	12.98 ± 2.10 b	8.36 ± 0.23 ab	7.76 ± 0.71 a
Malic	0.10 ± 0.00 a	2.60 ± 0.25 a	0.10 ± 0.00 a	9.15 ± 4.02 a
Citric	2.21 ± 0.50 a	2.54 ± 0.17 a	2.51 ± 0.23 a	2.28 ± 0.36 a
Lactic	0.41 ± 0.05 a	0.43 ± 0.09 a	0.34 ± 0.02 a	0.35 ± 0.06 a

The lowercase letters (a and b) indicate statistically significant differences between the samples. The statistical significance of the relative abundances was determined by Tukey’s post hoc test with *p* < 0.05.

**Table 3:** Effect of different treatments on fatty acid concentrations.

Fatty Acids (mg/gFW)	Control	Germanium	AMF	AMF + Germanium
Tetradecanoic (C14:0)	0.68 ± 0.05 a	0.78 ± 0.20 a	0.81 ± 0.08 b	1.29 ± 0.29 c
Pentadecanoic (C16:0)	14.96 ± 1.16 a	16.87 ± 1.70 b	16.68 ± 0.78 b	14.48 ± 2.15 a
Eicosanoic (C20:0)	0.84 ± 0.06 a	1.81 ± 0.33 b	0.90 ± 0.01 a	1.49 ± 1.81 ab
Docosanoic (C22:0)	0.96 ± 0.05 a	1.43 ± 0.05 b	1.07 ± 0.04 a	1.40 ± 0.10 b
Octadecanoic (C18:0)	7.71 ± 0.41 ab	11.45 ± 1.83 b	8.60 ± 0.33 ab	6.47 ± 1.09 a
Pentacosanoic (C24:0)	0.17 ± 0.00 a	0.14 ± 0.02 b	0.18 ± 0.00 b	0.14 ± 0.01 a
**Total saturated**	**25.33 ± 1.64 a**	**32.48 ± 2.34 b**	**28.20 ± 1.19 b**	**26.02 ± 4.51 a**
Pentadecanoic (C16:1)	0.90 ± 0.42 a	2.98 ± 0.62 b	1.43 ± 0.15 ab	1.91 ± 0.41 c
Pentadecanoic (C16:2)	0.48 ± 0.22 a	1.28 ± 0.15 b	0.54 ± 0.12 a	0.92 ± 0.20 c
Pentadecanoic (C16:3)	1.33 ± 0.13 a	1.36 ± 0.10 a	1.41 ± 0.04 a	1.58 ± 0.15 a
Octadecanoic (C18:1)	1.58 ± 0.06 a	5.95 ± 0.45 b	1.77 ± 0.06 a	5.42 ± 0.94 b
Octadecanoic (C18:2)	21.16 ± 0.47 ab	23.82 ± 0.80 b	23.60 ± 0.71 b	17.37 ± 2.40 a
Heptadecanoic (C18:3)	18.34 ± 0.38 a	34.40 ± 7.56 a	20.46 ± 0.61 a	21.48 ± 5.41 a
Heptadecanoic (C18:4)	1.02 ± 0.02 a	1.28 ± 0.15 a	1.13 ± 0.03 a	1.15 ± 0.09 a
Tetracosanoic (C20:3)	0.11 ± 0.01 a	0.36 ± 0.10 ab	0.11 ± 0.00 a	0.41 ± 0.05 b

The lowercase letters (a–c) indicate statistically significant differences between the samples. The statistical significance of the relative abundances was determined by Tukey’s post hoc test with *p* < 0.05.

**Table 4:** Effect of different treatments on antioxidant compounds.

Antioxidant	Phenol (mg/gFW)	Flavonoid (mg/gFW)	FRAP (mg/gFW)	ABTS %	DPPH %
Cont	9.75 ± 0.6 a	4.46 ± 0.1 a	8.16 ± 0.3 a	0.59 ± 0.1 a	9.59 ± 0.3 a
Germanium	23.86 ±1.2 b	6.72 ± 0.1 b	15.67 ± 1.6 b	1.45 ± 0.02 b	12.21 ± 1.1 a
AMF	10.18 ± 0.8 a	3.85 ± 0.1 a	3.55 ± 0.3 c	2.54 ± 0.03 c	54.75 ± 2.6 b
AMF + Germanium	20.91 ± 1.3 b	6.64 ± 0.2 b	7.60 ± 0.6 a	7.43 ± 0.3 d	69.64 ±2.5 b

The lowercase letters (a–d) indicate statistically significant differences between the samples. The statistical significance of the relative abundances was determined by Tukey’s post hoc test with *p* < 0.05.

**Table 5:** Effect of different treatments on antimicrobial activity.

Antibacterial Activity (Inhibation Zone Dimater (mm))	Control	Germanium	AMF	AMF + Germanium
*Staphylococcus saprophyticus*	19.47 ± 0.34 a	21.76 ± 1.24 a	19.92 ± 0.34 a	23.00 ± 1.25 a
*Staphylococcus epidermidis*	17.01 ± 0.06 a	18.17 ± 1.81 a	17.08 ± 0.06 a	19.89 ± 1.83 a
*Enterococcus faecalis*	17.05 ± 0.15 a	19.29 ± 1.16 a	17.31 ± 0.15 a	20.45 ± 1.17 a
*Streptococcus salivarius*	15.46 ± 0.39 a	19.58 ± 0.07 b	15.24 ± 0.39 a	19.79 ± 0.07 b
*Escherichia coli*	29.50 ± 1.15 a	18.71 ± 1.74 a	18.63 ± 1.16 a	20.37 ± 1.76 a
*Salmonella typhimurium*	18.51 ± 0.17 a	19.88 ± 1.55 a	18.80 ± 0.18 a	21.38 ± 1.56 a
*Pseudomonas aeruginosa*	24.33 ± 0.40 a	26.96 ± 1.97 a	24.87 ± 0.40 a	28.88 ± 1.99 a
*Proteus vulgaris*	20.97 ± 0.38 a	22.71 ± 1.55 a	21.46 ± 0.38 a	24.27 ± 1.59 a
*Enterobacter aerogenes*	22.26 ± 0.16 a	23.45 ± 1.60 a	22.56 ± 0.17 a	25.02 ± 1.61 a
*Serratia marcescens*	4.89 ± 0.04 a	4.37 ± 0.52 a	4.98 ± 0.05 a	4.86 ± 0.52 a
*Salmonella typhimurium*	12.53 ± 0.38 a	15.63 ± 0.79 bc	12.95 ± 0.38 ab	16.45 ± 0.80 c
*Candida albicans*	8.14 ± 0.16 a	9.31 ± 0.52 a	8.35 ± 0.16 a	9.84 ± 0.52 a
*Candida glabrata*	3.49 ± 0.05 a	3.85 ± 0.32 a	3.57 ± 0.05 a	4.16 ± 0.32 a
*Aspergillus flavus*	30.26 ± 0.32 a	40.22 ± 0.06 b	30.76 ± 0.33 a	40.47 ± 0.06 b

The lowercase letters (a–c) indicate statistically significant differences between the samples. The statistical significance of the relative abundances was determined by Tukey’s post hoc test with *p* < 0.05.

## Data Availability

Data are contained within the article.

## References

[1] Rosenberg E. (2007). Environmental speciation of germanium. Ecol. Chem. Eng..

[2] Goodman S. (1988). Therapeutic effects of organic germanium. Med. Hypoth..

[3] Lin K.F., Xu X.Q., Jin X., Ni H.Y., Xiang Y.L. (2005). Eco-toxicological effects of germanium stress on rice (*Oryza sativa* L.) and their critical value. Acta Ecol. Sin..

[4] Wang D.Z., Wang H., Li S.J., Cheng Z., Jin D. (2000). Effects of germanium on photosynthetic pigments of four species in microalgae. Acta Ecol. Sin..

[5] Guillard R.R.L., Murphy L.S., Foss P., Liaaen-Jensen S. (1985). *Synechococcus* spp. as likely zeaxanthin-dominant ultraphytoplankton in the North Atlantic. Limnol. Oceanogr..

[6] Bidigare R.R., Ondrusek M.E., Kennicutt II M.C., Iturriaga R., Harvey R., Hoham R.W., Macko S.A. (1993). Evidence a photoprotective for secondary carotenoids of snow algae. J. Phycol..

[7] Markham J.W., Hagmeier E. (1982). Observations on the effects of germanium dioxide on the growth of macro-algae and diatoms. Phycologia.

[8] Xie W.L., Yang P.H., Cai J.Y. (2010). Synthesis, characterization and antioxidation activity of germanium (IV)-quercetin complex. Chin. J. Anal. Chem..

[9] Bao Z.J., Ding Z.T., Cao Q.E., Zou Y.M. (2005). Free radical scavenging activity of rutin-germanium complex. Nat. Prod. Res. Dev..

[10] Provasoli L. (1968). Media and prospects for the cultivation of marine algae. Proceedings of the US-Japan Conference.

[11] Bonfante P., Genre A. (2010). Mechanisms underlying beneficial plant–fungus interactions in mycorrhizal symbiosis. Nat. Commun..

[12] Harley J.L., Smith S.E. (1983). Mycorrhizal Symbiosis.

[13] Giri B., Kapoor R., Mukerji K.G. (2007). Improved tolerance of *Acacia nilotica* to salt stress by arbuscular mycorrhiza, *Glomus fasciculatum* may be partly related to elevated K/Na ratios in root and shoot tissues. Microb. Ecol..

[14] Tisdall J.M., Oades J.M. (1982). Organic matter and water stable aggregates in soils. J. Soil Sci..

[15] Tigka T., Ipsilantis I. (2020). Effects of Sand Dune, Desert and Field Arbuscular Mycorrhizae on Lettuce (*Lactuca sativa* L.) Growth in a Natural Saline Soil. Sci. Hortic..

[16] Bona E., Cantamessa S., Massa N., Manassero P., Marsano F., Copetta A., Lingua G., D’Agostino G., Gamalero E., Berta G. (2017). Arbuscular mycorrhizal fungi and plant growth-promoting pseudomonads improve yield, quality and nutritional value of tomato: A field study. Mycorrhiza.

[17] Begum N., Qin C., Ahanger M.A., Raza S., Khan M.I., Ashraf M., Ahmed N., Zhang L. (2019). Role of arbuscular mycorrhizal fungi in plant growth regulation: Implications in abiotic stress tolerance. Front. Plant Sci..

[18] Schroeder H.A., Balassa J.J. (1967). Arsenic, germanium, tin, and vanadium in mice: Effects on growth, survival, and tissue levels. J. Nutr..

[19] Froelich P.N., Kaul W., Byrd J.T., Andreae M.O., Roe K.K. (1985). Arsenic, barium, germanium, tin, dimethylsulfide and nutrient biogeochemistry in Charlotte Harbor, Florida, a phosphorus-enriched estuary. Estuar. Coast. Shelf Sci..

[20] Cakmak I., Kurz H., Marschner H. (1995). Short-term effects of boron, germanium and high light intensity on membrane permeability in boron deficient leaves of sunflower. Physiol. Plant..

[21] Tang F., Ding X., Ding G., Liu D., He J., Shen J., Chu B. (2005). Effects of dioxide germanium on the protocorm of *Dendrobium officinale* in vitro. J. Nanjing Norm. Univ. Nat. Sci..

[22] Li Z.P., Zhu C.W., Wu P., Wang S.H., Sun Y.J. (2013). In vitro antioxidative activity of germanium-rich exopolysaccharides from *Ganoderm applanatum*. Modern Food Sci. Technol..

[23] Wei M., Yang C.Y., Jiang S.T. (2010). Effects of germanium on cell growth, polysaccharide production and cellular redox status in suspension cultures of protocorm-like bodies of *Dendrobium huoshanense*. Chin. J. Biotech..

[24] Lian Y.W., Duan P.C., Wang Y.L. (1999). The physiological effects of germanium dioxide on the germinating rice seeds. Chin. Bull. Bot..

[25] Xu J.S., Duan P.C., Wang Y.L., Lian Y.W. (1999). The effect of Ge and Si on the growth and development of rice. J. Xiamen Univ. Nat. Sci..

[26] Liu Y., Hou L., Zhao G., Li Q., Jiang Z. (2015). Mechanism and application of germanium in plant growth. Chin. J. Eco-Agric..

[27] Li X., Pan Y., Qi X., Zhang S., Zhi C., Meng H., Cheng Z. (2021). Effects of exogenous germanium and effective microorganisms on germanium accumulation and nutritional qualities of garlic (*Allium sativum* L.). Sci. Hortic..

[28] Bunea A., Andjelkovic M., Socaciu C., Bobis O., Neacsu M., Verhé R., Van Camp J. (2008). Total and individual carotenoids and phenolic acids content in fresh, refrigerated and processed spinach (*Spinacia oleracea* L.). Food Chem..

[29] Metha D., Belemkar S. (2014). Pharmacological activity of *Spinacia oleracea* Linn. A complete overview. Asian J. Pharm. Res. Dev..

[30] Llorach R., Martínez-Sánchez A., Tomás-Barberán F.A., Gil M., Ferreres F. (2008). Characterisation of polyphenols and antioxidant properties of five lettuce varieties and escarole. Food Chem..

[31] Nemadodzi L.E., Araya H., Nkomo M., Ngezimana W., Mudau N.F. (2017). Nitrogen, phosphorus, and potassium effects on the physiology and biomass yield of baby spinach (*Spinacia oleracea* L.). J. Plant Nutr..

[32] Roberts J.L., Moreau R. (2016). Functional properties of spinach (*Spinacia oleracea* L.) phytochemicals and bioactive. Food Funct..

[33] Hashem A., Kumar A., Al-Dbass A.M., Alqarawi A.A., Al-Arjani A.-B.F., Singh G., Farooq M., Allah E.F. (2019). Arbuscular mycorrhizal fungi and biochar improves drought tolerance in chickpea. Saudi J. Biol. Sci..

[34] Lichtenthaler H.K. (1987). Chlorophylls and Carotenoids: Pigments of Photosynthetic Biomembranes. Methods Enzymol..

[35] Wong K.H., Cheung P.C.K. (2000). Nutritional evaluation of some subtropical red and green seaweeds: Part I—Proximate composition, amino acid profiles and some physico-chemical properties. Food Chem..

[36] Lowry O.H., Rosebrough N.J., Farr A.L., Randall R.J. (1951). Protein measurement with the Folin phenol reagent. J. Biol. Chem..

[37] Shiva S., Enninful R., Roth M.R., Tamura P., Jagadish K., Welti R. (2018). An efficient modified method for plant leaf lipid extraction results in improved recovery of phosphatidic acid. Plant Methods.

[38] Lee S.C., Prosky L., Vries J.W.D. (1992). Determination of total, soluble, and insoluble dietary fiber in foods—Enzymatic-gravimetric method, MES-TRIS buffer: Collaborative study. J. AOAC Int..

[39] Abd Elgawad H., Peshev D., Zinta G., Van den Ende W., Janssens I.A., Asard H. (2014). Climate extreme effects on the chemical composition of temperate grassland species under ambient and elevated CO_2_: A comparison of fructan and non-fructan accumulators. PLoS ONE.

[40] Sinha A.K., Giblen T., Abd Elgawad H., De Rop M., Asard H., Blust R., De Boeck G. (2013). Regulation of amino acid metabolism as a defensive strategy in the brain of three freshwater teleosts in response to high environmental ammonia exposure. Aquat. Toxicol..

[41] Almuhayawi M.S., Al Jaouni S.K., Almuhayawi S.M., Selim S., Abdel-Mawgoud M. (2021). Elevated CO_2_ improves the nutritive value, antibacterial, anti-inflammatory, antioxidant and hypocholestecolemic activities of lemongrass sprouts. Food Chem..

[42] Kumpaisal R., Hashimoto T., Yamada Y. (1987). Purification and characterization of dihydrodipicolinate synthase from wheat suspension cultures. Plant Physiol..

[43] Ravanel S., Gakière B., Job D., Douce R. (1998). Cystathionine γ-synthase from Arabidopsis thaliana: Purification and biochemical characterization of the recombinant enzyme overexpressed in *Escherichia coli*. Biochem. J..

[44] Hamad I., Abd Elgawad H., Al Jaouni S., Zinta G., Asard H., Hassan S., Hegab M., Hagagy N., Selim S. (2015). Metabolic analysis of various date palm fruit (*Phoenix dactylifera* L.) cultivars from Saudi Arabia to assess their nutritional quality. Molecules.

[45] Almuhayawi M.S., Hassan A.H.A., Abdel-Mawgoud M., Khamis G., Selim S., Al Jaouni S.K., Abd Elgawad H. (2020). Laser light as a promising approach to improve the nutritional value, antioxidant capacity and anti-inflammatory activity of flavonoid-rich buckwheat sprouts. Food Chem..

[46] Farfan-Vignolo E.R., Asard H. (2012). Effect of elevated CO_2_ and temperature on the oxidative stress response to drought in *Lolium perenne* L. and *Medicago sativa* L.. Plant Physiol. Biochem..

[47] Dada F.A., Oyeleye S.I., Ogunsuyi O.B., Olasehinde T.A., Adefegha S.A., Oboh G., Boligon A.A. (2017). Phenolic constituents and modulatory effects of Raffia palm leaf (*Raphia hookeri*) extract on carbohydrate hydrolyzing enzymes linked to type-2 diabetes. J. Tradit. Complement. Med..

[48] Brouns F., Bjorck I., Frayn K.N., Gibbs A.L., Lang V., Slama G., Wolever T.M.S. (2005). Glycaemic index methodology. Nutr. Res. Rev..

[49] Puerner N.J., Siegel S.M., Siegel B.Z. (1990). The experimental phytotoxicology of germanium in relation to silicon. Water Air Soil Pollut..

[50] Wang X.J., Ruan X., Sun K.S., Yang L.H., Liu H.J., Yang B. (2007). Production of enriched germanium barley seedling. Food Sci..

[51] Hao Z., Xie W., Chen B. (2019). Arbuscular mycorrhizal symbiosis affects plant immunity to viral infection and accumulation. Viruses.

[52] Zhang L., Fan J., Ding X., He X., Zhang F., Feng G. (2014). Hyphosphere interactions between an arbuscular mycorrhizal fungus and a phosphate solubilizing bacterium promote phytate mineralization in soil. Soil Biol. Biochem..

[53] Parihar M., Rakshit A., Meena V.S., Gupta V.K., Rana K., Choudhary M., Tiwari G., Mishra P.K., Pattanayak A., Bisht J.K. (2020). The potential of arbuscular mycorrhizal fungi in C cycling: A review. Arch. Microbiol..

[54] Amaya-Carpio L., Davies F.T., Fox T., He C. (2009). Arbuscular mycorrhizal fungi and organic fertilizer influence photosynthesis, root phosphatase activity, nutrition, and growth of *Ipomoea carnea* ssp. Fistulosa. Photosynthetica.

[55] Miko U.F. (2011). Kirschbaum, Does Enhanced Photosynthesis Enhance Growth? Lessons Learned from CO_2_ Enrichment Studies. Plant Physiol..

[56] Thompson M., Gamage D., Hirotsu N., Martin A., Seneweera S. (2017). Effects of Elevated Carbon Dioxide on Photosynthesis and Carbon Partitioning: A Perspective on Root Sugar Sensing and Hormonal Crosstalk. Front. Physiol..

[57] Loewe A., Einig W., Shi L., Dizengremel P., Hampp R. (2000). Mycorrhiza formation and elevated CO_2_ both increase the capacity for sucrose synthesis in source leaves of spruce and aspen. New Phytol..

[58] Vijitrotai P., Vijitrotai N., Puttongsiri T. (2023). Proximate analysis, mineral and germanium of *Ganoderma lucidum* or Lingzhi powder by spray dry affecting in different strains and their maltodextrin. Int. J. Agric. Technol..

[59] AbdElgawad H., Avramova V., Baggerman G., Van Raemdonck G., Valkenborg D., Van Ostade X., Guisez Y., Prinsen E., Asard H., Van den Ende W. (2020). Starch biosynthesis contributes to the maintenance of photosynthesis and leaf growth under drought stress in maize. Plant Cell Environ..

[60] Madany M.M.Y., Saleh A.M., Habeeb T.H., Hozzeincd W.N., AbdElgawad H. (2020). Silicon dioxide nanoparticles alleviate the threats of broomrape infection in tomato by inducing cell wall fortification and modulating ROS homeostasis. Environ. Sci. Nano.

[61] Naudts K., Van den Berge V., Farfan E., Rose P., AbdElgawad H., Ceulemans R., Janssens I.A., Asard H., Nijs I. (2014). Future climate alleviates stress impact on grassland productivity through altered antioxidant capacity. Environ. Exp. Bot..

[62] Pepe A., di Baccio D., Magnani E., Giovannetti M., Sbrana C. (2022). Zinc and Iron Biofortification and Accumulation of Health Promoting Compounds in Mycorrhizal *Chorium intybus* L.. J. Soil Sci. Plant Nutr..

[63] Dang J.Z., Sun H.Y., Xu B.Q., Feng L.X. (2005). Determination of SOD and amino acids in Ge-enriched mycelia of *Ganoderma lucidum*. Lishizhen Med. Mater. Medica Res..

[64] Li G.Z., Xu Y.X., Zhang C.L. (2006). Studies on the law of translocation and accumulation of germanium in rice plant. J. Jilin Agric. Sci..

[65] Halperin S.J., Barzilay A., Carson M., Roberts C., Lynch J., Komarneni S. (1995). Germanium accumulation and toxicity in barley. J. Plant Nutr..

[66] Shtark O., Puzanskiy R., Avdeeva G., Yemelyanov V., Shavarda A., Romanyuk D., Kliukova M., Kirpichnikova A., Tikhonovich I., Zhukov V. (2021). Metabolic alterations in *Pisum sativum* roots during plant growth and arbuscular mycorrhiza development. Plants.

[67] Sheteiwy M.S., El-Sawah A.M., Korany S.M., Alsherif E.A., Mowafy A.M., Chen J., Jośko I., Selim S., AbdElgawad H. (2022). Arbuscular Mycorrhizal Fungus “*Rhizophagus irregularis*” impacts on physiological and biochemical responses of ryegrass and chickpea plants under beryllium stress. Environ. Pollut..

[68] Versieren L., Evers S., AbdElgawad H., Asard H., Smolders E. (2017). Mixture toxicity of copper, cadmium, and zinc to barley seedlings is not explained by antioxidant and oxidative stress biomarkers. Environ. Toxicol. Chem..

[69] Huda-faujan N., Zubairi S.I., Baker A.A. (2023). Nutritional and Bioactive Constituents of Antioxidant and Antimicrobial Properties in *Spinacia oleracea*: A Review. Sains Malays..

[70] Kadam K.S., Gokhale N.S., Hugar S.M., Kohli N., Dodamani S., Tendulkar S. (2023). Comparative evaluation of antibacterial efficacy of *Chlorhexidine mouthwash* and *Momordica charantia*, *Spinacia oleracea* mouthwash against *Streptococcus mutans*, *Lactobacillus* spp., and *Porphyromonas gingivalis*—An in vitro study. J. Orofac. Sci..

